# Transcriptional regulation of the human, porcine and bovine OCTN2 gene by PPARα via a conserved PPRE located in intron 1

**DOI:** 10.1186/s12863-014-0090-y

**Published:** 2014-09-08

**Authors:** Huidi Luo, Yuanqing Zhang, Huihui Guo, Li Zhang, Xi Li, Robert Ringseis, Gaiping Wen, Dequan Hui, Aihua Liang, Klaus Eder, Dongchang He

**Affiliations:** 1 Institute of Animal Husbandry and Veterinary Medicine, Shanxi Provincial Academy of Agricultural Sciences, Taiyuan 030031, P. R. China; 2 Institute of Animal Nutrition and Nutrition Physiology, Justus-Liebig-University Giessen, Giessen, 35392, Germany; 3 Key Laboratory of Chemical Biology and Molecular Engineering of Ministry of Education, Institute of Biotechnology, Shanxi University, Taiyuan 030006, P. R. China

**Keywords:** OCTN2, PPARα, Pig, Cattle, Human

## Abstract

**Background:**

The novel organic cation transporter 2 (OCTN2) is the physiologically most important carnitine transporter in tissues and is responsible for carnitine absorption in the intestine, carnitine reabsorption in the kidney and distribution of carnitine between tissues. Genetic studies clearly demonstrated that the mouse OCTN2 gene is directly regulated by peroxisome proliferator-activated receptor α (PPARα). Despite its well conserved role as an important regulator of lipid catabolism in general, the specific genes under control of PPARα within each lipid metabolic pathway were shown to differ between species and it is currently unknown whether the OCTN2 gene is also a PPARα target gene in pig, cattle, and human. In the present study we examined the hypothesis that the porcine, bovine, and human OCTN2 gene are also PPARα target genes.

**Results:**

Using positional cloning and reporter gene assays we identified a functional PPRE, each in the intron 1 of the porcine, bovine, and human OCTN2 gene. Gel shift assay confirmed binding of PPARα to this PPRE in the porcine, bovine, and the human OCTN2 gene.

**Conclusions:**

The results of the present study show that the porcine, bovine, and human OCTN2 gene, like the mouse OCTN2 gene, is directly regulated by PPARα. This suggests that regulation of genes involved in carnitine uptake by PPARα is highly conserved across species.

## 1 Background

The peroxisome proliferator-activated receptor α (PPARα) is a ligand-activated transcription factor and serves as an important regulator of lipid metabolism and energy homeostasis [1]–[4]. Ligands of PPARα are fatty acids which are released from white adipose tissue during energy deprivation and taken up into tissues during this state or exogenous ligands such as fibrates (e.g., WY-14,643, clofibrate) [5]. Transcriptional regulation of genes by PPARα is mediated by binding of activated PPAR/retinoid X receptor (RXR) heterodimers to specific DNA sequences, called peroxisome proliferator response elements (PPRE), thereby stimulating the expression of those genes. The PPRE consists of a direct repeat of two copies of a AGGTCA-like sequence separated by a single base, commonly called Direct Repeat 1 (DR1), and has been identified in the promoter, intron and 5’-untranslated region of PPAR target genes [6]–[10].

Interestingly, several earlier studies repeatedly reported that energy deprivation and treatment with fibrates causes a strong, up to five-fold elevation in the concentration of carnitine in the liver of rats [11]–[13]. Carnitine plays an important role in lipid and energy metabolism by acting as a shuttling molecule for the translocation of long-chain fatty acids from the cytosol into the mitochondrial matrix, where β-oxidation occurs. The mechanism underlying this phenomenon remained obscure until it was demonstrated many years later that activation of hepatic PPARα, which is induced by both energy deprivation and fibrate treatment, causes an up-regulation of the novel organic cation transporter 2 (OCTN2) in liver cells [14]. OCTN2 is the physiologically most important carnitine transporter facilitating carnitine absorption in the intestine, carnitine reabsorption in the kidney, and carnitine distribution between tissues [15]. In subsequent experiments, convincing evidence for the PPARα-dependency of this effect could be provided by demonstrating that the energy deprivation- or fibrate-induced increase in hepatic carnitine concentration and up-regulation of OCTN2 occurs only in wild-type mice but not in PPARα knockout mice [16],[17]. Moreover, it could be shown recently that the mouse gene encoding OCTN2 contains a functional PPRE in intron 1 and is therefore directly activated by PPARα [8].

Besides the abovementioned studies in rats and mice, studies in pigs [18],[19], chicken [20] and cattle [21],[22] demonstrated that PPARα activation increases hepatic or cellular carnitine content and up-regulates OCTN2 in the liver or hepatocytes, suggesting that the effect of PPARα as a regulator of carnitine uptake is well conserved across species [23]. Despite the highly conserved role of PPARα as an important regulator of lipid metabolism in general, the specific genes under control of PPARα within each lipid metabolic pathway were shown to differ between species [24], and it is currently unknown whether the OCTN2 gene is also a PPARα target gene in pig, cattle and human. Comparative sequence analysis revealed that the functional PPRE identified in the mouse OCTN2 intron 1 [8] is completely identical (100%) to a putative PPRE present in the porcine, bovine and human OCTN2 intron 1 [23] indicating a high degree of conservation of this sequence and a similar regulation of the OCTN2 gene across these species. However, the functionality of a PPRE cannot be predicted from sequence comparison of closely related species with 100% certainty but rather has to be thoroughly established by cell culture, reporter gene and gel shift experiments. Based on this we hypothesized that the porcine, bovine, and human OCTN2 gene, like the mouse OCTN2 gene, are directly regulated by PPARα. In this study we therefore performed *in silico*-analyses and reporter gene and gel shift assays to identify a functional PPRE in the porcine, bovine, and human OCTN2 gene.

## 2 Results and discussion

**Figure 1:**
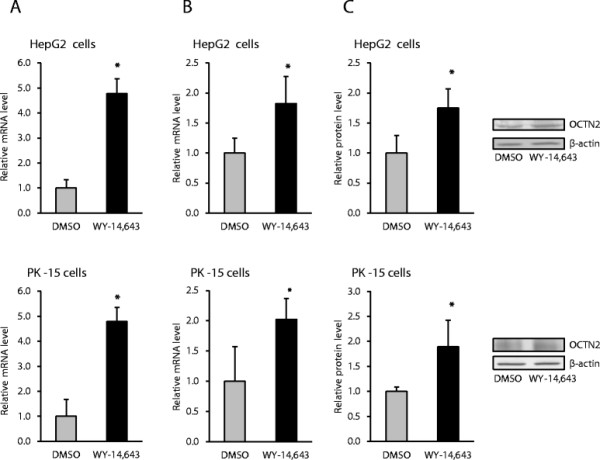
**Activation of PPARα and stimulation of OCTN2 gene expression by PPARα agonist WY-14,643 in human and porcine cells.** Human HepG2 cells and porcine PK-15 cells were treated without or with 150 μM WY-14,643 for 24 h to study the effect on CPT1 mRNA level **(A)**, OCTN2 mRNA level **(B)** and OCTN2 protein level **(C)**. Representative immunoblots for OCTN2 and β-actin are shown. Bars represent means ± SD for three independent experiments. *Different from DMSO-treated control, P < 0.05.

To study regulation of OCTN2 gene expression by PPARα in porcine and human cells, we treated porcine PK-15 cells and human HepG2 cells without or with PPARα agonist WY-14,643. Treatment of both cell lines with WY-14,643 increased mRNA levels of the known PPARα target gene CPT1 about 5-fold indicating successful activation of PPARα (Figure 1A). Relative mRNA and protein levels of OCTN2 were also elevated by WY-14,643 about 2-fold both, in porcine PK-15 and human HepG2 cells (Figure 1B and C) indicating that porcine and human OCTN2 are regulated directly by PPARα in pigs and humans. Like in porcine and human cells, we have recently shown that mRNA and protein levels of OCTN2 are increased by the PPARα agonist WY-14,643 about 2.2- and 1.4-fold, respectively, in bovine MDBK cells and co-treatment of MDBK cells with a PPARα antagonist abolished the effect of WY-14,643 [22] suggesting that bovine OCTN2 is probably also regulated directly by PPARα.

**Figure 2:**
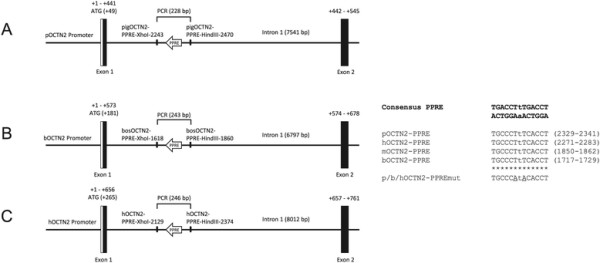
**Schematic illustration of the PPRE within the intron 1 of porcine, bovine and human OCTN2 gene.** Schematic illustration of the PPRE within the intron 1 of porcine **(A)**, bovine **(B)**, and human **(C)** OCTN2 gene. Position of putative PPRE and the primers used for reporter gene construction are indicated. Comparison of PPRE from porcine, bovine, and human OCTN2 with PPRE from mouse OCTN2 and consensus PPRE sequence is shown. Matching nucleotides between human, mouse, porcine and bovine OCTN2 are shown by asterisks.

To provide evidence for direct regulation of OCTN2 by PPARα, we next performed *in silico*-analysis of intron 1 of the porcine, bovine, and human OCTN2 gene using DNA sequences from NCBI Genbank (Accession number XM_003123912 for porcine cDNA, CU372899 for porcine genomic DNA; NM_001046502 for bovine cDNA, AC14966 for bovine genomic DNA; NM_003060 for human cDNA, AC000137 for human genomic DNA) and NUBIScan software (nuclear receptor binding site scanner; [25]). According to this, we observed one putative PPRE, which had 100% sequence identity with the functional PPRE of the mouse OCTN2 intron 1 (located at 1850 to 1862), each located in the intron 1 of porcine OCTN2 (at position nt 2329 to 2341; Figure 2A), bovine OCTN2 (at position nt 1717 to 1729; Figure 2B), and human OCTN2 (at position nt 2271 to 2283; Figure 2C). The porcine, bovine, and human putative PPRE displayed extreme low p-values (0.00129, 0.00106, and 0.0007, respectively), indicating that these regions are likely candidates for mediating PPARα-dependent regulation of porcine, bovine, and human OCTN2.

**Figure 3:**
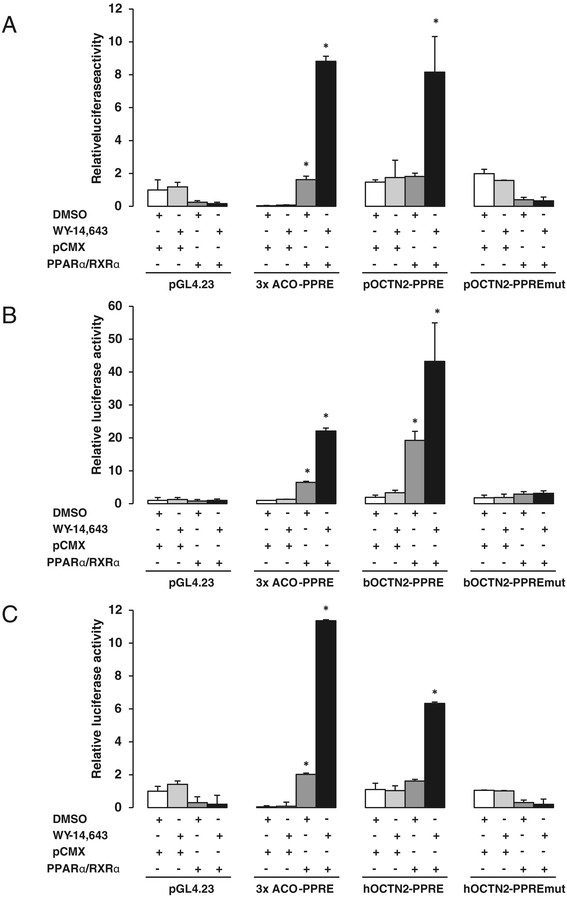
**Activation of porcine, bovine and human OCTN2-PPRE reporter gene constructs by PPARα.** HepG2 cells were transfected with either **(A)** wild-type plasmid pGL4.23-pOCTN2-PPRE-Luc or mutant plasmid pGL4.23-pOCTN2-PPREmut-Luc or **(B)** wild-type plasmid pGL4.23-bOCTN2-PPRE-Luc or mutant plasmid pGL4.23-bOCTN2-PPREmut-Luc or **(C)** wild-type plasmid pGL4.23-hOCTN2-PPRE-Luc or mutant plasmid pGL4.23-hOCTN2-PPREmut-Luc. Cells were co-transfected with pCMX-mPPARα and pCMX-mRXRα or pCMX (empty vector) expression plasmids, and pGL4.74-RLuc as internal control and normalization plasmid. The plasmids pGL4.23-luc and 3xACO-PPRE were used as negative and positive control plasmids, respectively. 12 h after transfection cells were cultured in medium in the presence or absence of 50 μM WY-14,643 for 24 h and, subsequently, luciferase activities determined by luminometry. Bars represent means ± SD for one out of three independent experiments each performed in triplicate. The other experiments revealed similar results. *Different from control (empty vector pCMX), P < 0.05.

To further examine whether the identified putative PPRE are functional, we generated luciferase reporter gene constructs containing a 228 bp (porcine), a 243 bp (bovine) and a 246 bp (human) fragment, each spanning the putative PPRE of the OCTN2 intron 1 in front of a luciferase gene, by means of positional cloning. Subsequently, we transiently transfected these constructs into HepG2 cells and tested their responsiveness to PPARα/RXRα co-expression and PPARα agonist WY-14,643 in dual-luciferase reporter assays. As shown in Figure 3A-C, the luciferase activity of all OCTN2-PPRE constructs was increased dramatically by co-expression of exogenous mouse PPARα/RXRα and treatment with WY-14,643 (8-fold for the porcine OCTN2-PPRE construct, 43-fold for the bovine OCTN2-PPRE construct, and 6-fold for the human OCTN2-PPRE construct) compared to empty vector. The markedly stronger response of the bovine OCTN2 construct compared to the human and porcine OCTN2 constructs should not be over-interpreted and may be explained by a lower binding of transcriptional repressors to the bovine construct compared to the other constructs. In contrast HepG2 cells transfected with the mutant OCTN2-PPRE constructs, in which the putative PPRE were selectively mutated, resulted in a complete loss of responsiveness to exogenous PPARα/RXRα and WY-14,643 (Figure 3A-C). These findings indicated that the putative PPRE in the OCTN2 intron 1 is a critical element responsible for PPARα-dependent regulation of the porcine, bovine and human OCTN2 gene.

**Figure 4:**
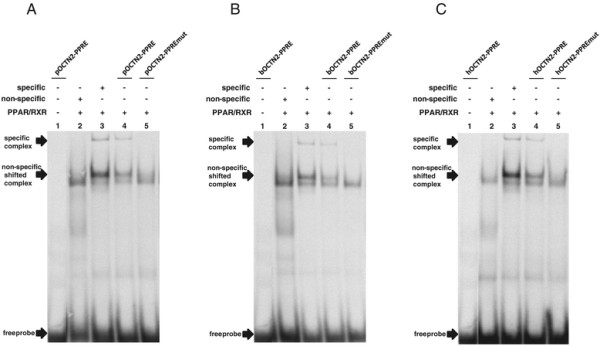
**Binding of*in vitro*translated mouse PPARα/RXRα to the PPRE in the intron 1 of porcine, bovine and human OCTN2 gene.** EMSA was performed using DIG-labeled oligonucleotides corresponding to the wild-type and the mutated PPRE of porcine **(A)**, bovine **(B)**, and human **(C)** OCTN2 and *in vitro*-translated mouse PPARα/RXRα. DIG-labeled specific and non-specific probes were used as positive and negative controls, respectively. Arrows indicate DNA/protein complexes and free DNA probes.

To finally confirm that this PPRE is functional, we studied *in vitro*-binding of the PPARα/RXRα heterodimer to this PPRE using *in vitro*-translated PPARα/RXRα and labeled double-stranded oligonucleotides containing this PPRE sequence in gel shift assays (EMSA). As shown in Figure 4A-C, in the presence of PPARα/RXRα proteins, a band appeared representing a complex between the labeled oligonucleotide and the PPARα/RXRα heterodimer (lane 4, Figure 4A-C). On the contrary, no band for the DNA-PPARα/RXRα complex was observed when an oligonucleotide with a mutation in the putative PPRE was used (lane 5, Figure 4A-C). These observations indicated that the PPARα/RXRα heterodimer binds specifically to the identified PPRE in the intron 1 of porcine, bovine, and human OCTN2 gene, and this PPRE contributes to PPARα-dependent regulation of the OCTN2 gene in these species.

We have recently postulated that the physiological meaning of PPARα-mediated up-regulation of the OCTN2 gene is to supply cells with sufficient carnitine required for transport of excessive amounts of fatty acids into the mitochondrion, and therefore plays an important role in the adaptive response of cells to PPARα activation [14]. In cattle and pigs, PPARα activation physiologically occurs in the liver during early lactation and peak lactation, respectively, because the negative energy balance during these states leads to the release of fatty acids from adipose tissues which are taken up into the liver and bind to and activate PPARα [26]–[28]. Thus, the observation from the present study that the porcine and bovine OCTN2 gene is regulated by PPARα provides a plausible explanation for the recent finding that OCTN2 in the liver is up-regulated during early lactation in high-producing dairy cows [21] and during peak lactation in sows [28]. Our observation that the human OCTN2 gene is regulated by PPARα may be not only relevant with regard to carnitine homeostasis but also to tissue distribution and intestinal intestinal absorption of various other compounds, because OCTN2 is polyspecific and able to bind other monovalent cations and various drugs such as verapamil, spironolactone or mildronate [29]–[31].

## 3 Conclusions

The present results show that the intron 1 of porcine, bovine, and human OCTN2 gene contains a functional PPRE which is critical for PPARα-dependent regulation of the OCTN2 gene. This indicates that the porcine, bovine, and human OCTN2 gene, like the mouse OCTN2 gene [8], are directly regulated by PPARα. Given the fundamental role of OCTN2 for intestinal carnitine absorption and renal carnitine reabsorption and therefore carnitine homeostasis, our findings suggest that PPARα is a key regulator of carnitine homeostasis in these species. The similar regulation of the OCTN2 gene by PPARα between mouse, pig, cattle, and human suggests that regulation of genes involved in carnitine uptake by PPARα is highly conserved across species.

## 4 Methods

### 4.1 Chemicals

RPMI1640 GlutaMax-1 medium, fetal calf serum and gentamycin were obtained from Invitrogen. WY-14,643 was purchased from Sigma (Beijing, China).

### 4.2 Cell culture

The human hepatoma cell line HepG2 was obtained from Boster Biological Technology, Ltd. (Wuhan, China). The porcine kidney cell line PK-15 was purchased from Cell Lines Service GmbH (Eppelheim, Germany). HepG2 cells were grown in RPMI1640 GlutaMax-1 medium and PK-15 cells were cultivated in Hy-Clone Minimum Essential Medium/Earle’s Balanced Salt Solution (MEM/EBSS) medium. The media were supplemented with 10% fetal bovine serum and 0.05 mg/mL gentamicin. All cells were maintained at 37°C in a humidified atmosphere of 95% air and 5% CO_2_. Media were changed every 2 days. After reaching a confluence of 70–80%, the cells were either sub-cultivated or used for experiments.

### 4.3 RNA Isolation and qPCR analysis

For qPCR experiments, cells were seeded in 24-well culture plates at a cell density of 0.7 × 10^5^ (PK-15) and 1 × 10^5^ (HepG2 cells) per well. After reaching confluence, cells were treated for 24 h with either 150 μM of the PPARα agonist WY-14,643 or DMSO as vehicle control in low-serum medium (0.5% FCS) for HepG2 cells or in medium without serum but with 5 mg/L bovine insulin for PK-15 cells. Following treatment, total RNA was extracted from cells using Trizol™ reagent according to the manufacturer’s protocol. cDNA synthesis and qPCR using gene-specific primers were performed as described recently in detail [32]. Gene-specific primers were synthesized by TaKaRa (Dalian, China) and are listed in Table 1. Relative changes in gene expression were calculated using the 2^−ΔΔCT^ method [33] using GAPDH as internal control. Normalized gene expression ratio of WY-14,643 treated cells was presented relative to that of DMSO-treated control cells, which was set to 1.0.

**Table 1:** Gene-specific primers used for qPCR

**Gene name**	**Oligonucleotide sequences (forward, reverse)**	**PCR product size (bp)**	**Accession number**
pOCTN2	TGACCATATCAGTGGGCTA, AGTAGGGAGACAGGATGCT	384	XM_003123912
hOCTN2	GACCATATCAGTGGGCTATTT, CTGCATGAAGAGAAGGACAC	199	NM_003060
pGAPDH	AGGGGCTCTCCAGAACATCATCC, TCGCGTGCTCTTGCTGGGGTTGG	446	AF017079
hGAPDH	GCCTTCCGTGTCCCCACTGC, CAATGCCAGCCCCAGCGTCA	211	NM_002046
pCPT1	GCATTTGTCCCATCTTTCGT, GCACTGGTCCTTCTGGGATA	198	AF288789
hCPT1	TCACCTCTTCTGCCTTTACG, AGTCAAACAGCTCCACTTGC	132	NM_001876

*Abbreviations: b* bovine, *h* human, *p* porcine.

### 4.4 Western blot analysis

For western blot experiments, cells were seeded in 6-well culture plates at a cell density of 2 × 10^5^ (PK-15 cells) and 5 × 10^5^ (HepG2 cells) per well. After reaching confluence, cells were treated as described for qPCR experiments. Following treatment, cells were harvested and lysed with RIPA lysis buffer (50 mM Tris pH 7.5, 150 mM NaCl, 1 mM EDTA, 1% Triton X-100, 0.1% SDS, 1% sodium deoxycholate) containing protease inhibitors (Sigma-Aldrich, Steinheim, Germany). Protein concentrations in cell lysates were determined by the BCA protein assay (Interchim, Montlucon, France). SDS-PAGE was performed with a 4% stacking gel and a 10% resolving gel. Afterwards proteins were transferred to a nitrocellulose membrane. The membranes were blocked overnight at 4°C in blocking solution (5% skim milk in Tris buffered saline with Tween-20), and then incubated with mouse monoclonal β-actin (1:500, Abcom, Cambridge, UK) or mouse polyclonal OCTN2 (1:500, Abcom, Cambridge, UK) primary antibodies for 2 h at room temperature and overnight at 4°C, respectively. The membranes were washed with TBS-T (50 mmol/L Tris, 150 mmol/L NaCl, pH 7.5, 0.2% Tween-20) and incubated with a horseradish peroxidase conjugated secondary monoclonal anti-mouse-IgG antibody (1:5000, Jackson Immuno Research, Suffolk, UK) for 1 h at room temperature. The blots were developed by using the Amersham™ ECL Plus Western Blotting Detection System (GE Healthcare, Munich, Germany) and detected by a chemiluminescence imager (Syngene, Cambridge, UK). The signal intensities of specific bands were detected with a Bio-Imaging system (Syngene, Cambridge, UK) and quantified using Syngene GeneTools software. For calculation of protein expression levels, the band intensity of OCTN2 was normalized by that of β-actin and normalized protein expression level of WY-14,643 treated cells was presented relative to that of DMSO-treated control cells, which was set to 1.0.

### 4.5 Isolation of porcine and bovine genomic DNA

50**–**100 mg ear tissues of pig and cattle were obtained from Mashen pig and Jinnan yellow cattle of Shanxi. These experiments were approved by the Shanxi Administration Office of Laboratory Animal. The tissues were washed with 1× PBS followed by incubation in 500 μL lysis buffer containing 100 mM Tris–HCl, 5 mM EDTA, 0.2% SDS, 200 mM NaCl and 100 μg/mL proteinase K at 55°C overnight. At the next day, the lysate was centrifuged for 5 min and the supernatant was transferred into a new tube. Subsequently, 500 μL of isopropanol was added and centrifuged again for 20 min. Finally, DNA pellets were washed with 70% ethanol and suspended in TE buffer.

### 4.6 Isolation of human genomic DNA

For isolation of human genomic DNA, HepG2 cells were seeded in 6-well culture plates at a cell density of 1 × 10^6^ per well. After reaching 70–80% confluence the medium was removed and the cells were washed with 1× PBS. Following, cells were collected by scraping and suspended in 500 μL lysis buffer containing 5% SDS, 250 mM Tris and 5 N NaOH. Subsequently, an equal volume of a 1:1 (v:v) mixture of phenol/chloroform was added and mixed. After a 15 min centrifugation step, the upper aqueous phase was transferred into a new tube containing 100% ethanol and centrifuged again for 10 min. Finally, DNA pellets were washed with 70% ethanol and resuspended in TE buffer.

### 4.7 Construction of the reporter genes and site-directed mutagenesis

A 228 bp DNA fragment spanning 2243 to 2470 in intron 1 of porcine (p) OCTN2 and a 243 bp DNA fragment spanning 1618 to 1860 in intron 1 of bovine (b) OCTN2, each containing a putative PPRE, were amplified by PCR using genomic DNA isolated from Mashen pigs and Jinnan yellow cattle of Shanxi. In addition, a 246 bp DNA fragment spanning 2129 to 2374 in intron 1 of the human (h) OCTN2 gene containing a putative PPRE was amplified by PCR using human genomic DNA isolated from HepG2 cells. Specific primers containing XhoI and HindIII sites for PCR amplification were synthesized by TaKaRa and are listed in Table 2. Each PCR fragment was cloned into the XhoI and HindIII site of the pGL4.23 plasmid (Shanghai Promega Biological Products, Ltd. Shanghai, China) which contains the minimal promoter miniP in front of the luciferase reporter gene luc2. The generated constructs (pGL4.23-pOCTN2-PPRE, pGL4.23-bOCTN2-PPRE, and pGL4.23-hOCTN2-PPRE) were sequenced to confirm absence of any mutation. The mutant luciferase reporter plasmids (pGL4.23-pOCTN2-PPREmut, pGL4.23-bOCTN2-PPREmut, and pGL4.23-hOCTN2-PPREmut) were generated using Site-Directed Mutagenesis kit according to the manufacturer’s protocol (Agilent Technologies Co. Ltd, Beijing, China). Primer sequences used for mutagenesis are shown in Table 2. The mutant constructs were controlled by DNA sequencing.

**Table 2:** Oligonucleotides used for cloning, mutagenesis and EMSA

**Denomination of oligonucleotides**	**Oligonucleotide sequences**	**PCR product size (bp)**	**Accession number**
Oligos used for cloning			
pOCTN2-PPRE-HindIII	ATAAAGCTTCAGCCTCTCTGTTTCGTCAG	228	CU372899
pOCTN2-PPRE-XhoI	ATACTCGAGGAGCTATGTTGCTGCCAGTG		
bOCTN2-PPRE-HindIII	ATAAAGCTTCAGAAGGGTCCTTGAGCTAT	243	AC149665
bOCTN2-PPRE-XhoI	ATACTCGAGCCAACAGTGACTGTTCACCA		
hOCTN2-PPRE-HindIII	ATAAAGCTTGCTCTGAACTTCAAGTCAAGC	246	AC000137
hOCTN2-PPRE-XhoI	ATACTCGAGCTGAGTGATGGTGGCATTGA		
Oligos used for mutagenesis and EMSA-mut			
pOCTN2-PPREmut-For	AACCTGTAAGTAGGTGTATGGGCACACAACTCGTA		
pOCTN2-PPREmut-Rev	TACGAGTTGTGTGCCCATACACCTACTTACAGGTT		
bOCTN2-PPREmut-For	AACCTGGAAGTAGGTGTATGGGCACAGAGCTCTTT		
bOCTN2-PPREmut-Rev	AAAGAGCTCTGTGCCCATACACCTACTTCCAGGTT		
hOCTN2-PPREmut-For	AACATATAAGTAGGTGtAtGGGCACATAACTCCTT		
hOCTN2-PPREmut-Rev	AAGGAGTTATGTGCCCaTaCACCTACTTATATGTT		
Oligos used for EMSA			
pOCTN2-PPRE-For	AACCTGTAAGTAGGTGAAAGGGCACACAACTCGTA		
pOCTN2-PPRE-Rev	TACGAGTTGTGTGCCCTTTCACCTACTTACAGGTT		
bOCTN2-PPRE-For	AACCTGGAAGTAGGTGAAAGGGCACAGAGCTCTTT		
bOCTN2-PPRE-Rev	AAAGAGCTCTGTGCCCTTTCACCTACTTCCAGGTT		
hOCTN2-PPRE-For	AACATATAAGTAGGTGAAAGGGCACATAACTCCTT		
hOCTN2-PPRE-Rev	AAGGAGTTATGTGCCCTTTCACCTACTTATATGTT		
EMSA-sp.-For	TCTTCCCGAACGTGACCTTTGTCCTGGTCCCCTTT		
EMSA-sp.-Rev	TCAAAGGGGACCAGGACAAAGGTCACGTTCGGGAA		
EMSA-non-sp.-For	TTCCCATCTTGTGAGCTGTCACCCATGGTGGGGTG		
EMSA-non-sp.-Rev	CACCCCACCATGGGTGACAGCTCACAAGATGGGAA		

*Abbreviations: b* bovine, *h* human, *p* porcine.

### 4.8 Transient transfection and dual-luciferase reporter assay

Transient transfection was carried out as described previously [6]. Briefly, HepG2 cells were grown in RPMI1640 GlutaMax-1 medium supplemented with 10% fetal calf serum and 0.05 mg/mL gentamycin. After reaching 70–80% confluency, cells were seeded in 96-well culture plates at a cell density of 4.5 × 10^4^ per well. Then cells were transiently transfected with 50 ng of the generated reporter gene constructs and co-transfected with 50 ng of both, mouse PPARα expression plasmid (pCMX-mPPARα) and mouse RXRα expression plasmid (pCMX-mRXRα) or 100 ng empty vector (pCMX) using FuGENE 6 transfection reagent (Promega) according to the manufacturer’s protocol. Cells were co-transfected with 5 ng pGL4.74 plasmid (Promega) encoding the renilla luciferase reporter gene as internal control reporter vector to normalize for differences in transfection efficiency. A plasmid containing three copies of the functional PPRE from the acyl-CoA oxidase (ACO) promoter in front of a luciferase reporter gene (3xACO-PPRE plasmid) and the empty vector pGL4.23 were transfected as positive and negative controls, respectively. 12 h after transfection, the cells were treated with either 50 μM WY-14,643 to achieve activation of PPARα or vehicle only (DMSO = control) for 24 h. Luciferase activities were determined with the Dual-Luciferase Reporter Assay System from Promega according to the manufacturer’s instructions using a Mithras LB940 luminometer (Berthold Technologies, Bad Wildbad, Germany). For control of background luminescence Firefly- and Renilla-luciferase activities were also determined in the lysates of nontransfected control cells and substracted from total luminescence of transfected cells. Data were normalized for transfection efficiency by dividing Firefly-luciferase activity of the generated reporter constructs by that of Renilla-luciferase activity of the cotransfected pGL4.74 Renilla luciferase plasmid. Results represent normalized luciferase activities and are shown relative to cells transfected with the empty vector pGL4.23 and treated with DMSO only which was set to 1.

### 4.9 Electrophoretic mobility shift assay (EMSA)

EMSA was performed using DIG Gel Shift Kit, 2nd (Roche) according to the manufacturer’s protocol. An oligonucleotide containing the rat ACO-PPRE and a random oligonucleotide were used as specific and non-specific controls, respectively. Synthesized wild-type and mutated oligonucleotides used for EMSA are listed in Table 1. The mouse PPARα and RXRα proteins were generated from the expression vectors by *in vitro* transcription/translation using TNT® Quick Coupled Transcription/Translation Kit (Promega) according to the manufacturer’s protocol. The DNA-protein complexes were detected by chemiluminescence using Anti-Digoxigenin-AP Conjugate and CSPD (Roche) and quantified using a Bio-Imaging system (Syngene, Cambridge, UK) and Syngene GeneTools software.

### 4.10 Statistical analysis

Quantitative data are expressed as mean ± standard deviation (SD) based on at least three independent experiments performed in triplicate. Differences were analysed by one-way ANOVA using SAS 9.1.3 (SAS Institute, Inc., 2001). A P-value < 0.05 was considered to be significant.

## Competing interests

The authors declare that they have no competing interests.

## Authors’ contributions

HL, YZ, HG, LZ, XL, and GW carried out the molecular biological studies, participated in the sequence alignment and drafted the manuscript. GW and DH participated in the sequence alignment. HL, YZ, GW, DH, and AL participated in the design of the study and performed the statistical analysis. RR, GW, KE, and DoH conceived of the study, and participated in its design and coordination and helped to draft the manuscript. All authors read and approved the final manuscript.
